# Roles of efflux pumps and nitroreductases in metronidazole-resistant *Trichomonas vaginalis*

**DOI:** 10.1007/s00436-025-08463-7

**Published:** 2025-02-12

**Authors:** Ana Paunkov, Doris Strasser, Philipp Huber, David Leitsch

**Affiliations:** Institute for Specific Prophylaxis and Tropical Medicine, Center for Pathophysiology, Infectiology, and Immunology, Medical University of Vienna, Kinderspitalgasse 15, 1090 Vienna, Austria

**Keywords:** *Trichomonas vaginalis*, Metronidazole, Efflux pump inhibitors, Nitroreductase, Antimicrobial resistance, Iron reduction

## Abstract

*Trichomonas vaginalis* infections significantly impact public health and are associated with increased likelihood of HIV infection, prostate cancer, and pregnancy complications. Current treatment relies almost exclusively on 5-nitroimidazoles, particularly metronidazole, raising concerns about drug resistance and treatment efficacy. This study is aimed at evaluating the effectiveness of metronidazole and tinidazole on metronidazole-resistant strains of *T. vaginalis* and at determining whether efflux pump inhibitors could reverse metronidazole resistance. Additionally, the roles of nitroreductases in metronidazole resistance were also studied. Metronidazole and tinidazole were tested on both metronidazole-sensitive and -resistant *T. vaginalis* strains. A checkerboard assay was conducted to assess the potential synergy between metronidazole or tinidazole and efflux pump inhibitors. Nitroreductase activity and ferric iron reduction assays were employed to study the functions of nitroreductases. Tinidazole demonstrated better effectiveness against metronidazole-resistant strains compared to metronidazole, with lower minimal lethal concentration levels. However, the tested efflux pump inhibitors did not significantly enhance the efficacy of metronidazole or tinidazole. Pyrimethamine showed some activity but did not improve the efficacy of the 5-nitroimidazoles in combination. Investigations into the role of nitroreductases and other enzymes in metronidazole resistance revealed no clear downregulation trend in resistant strains. Notably, nitroreductase 8 was capable of reducing ferric iron. While tinidazole remains a viable alternative for treating metronidazole-resistant *T. vaginalis*, efflux pump inhibitors do not effectively reverse resistance. The identification of nitroreductase’s 8 iron-reducing activity suggests its involvement in metronidazole resistance mechanisms. This finding highlights the need for continued research to develop new treatment strategies and improve the management of trichomoniasis, ultimately reducing its public health burden.

**Supplementary Information:**

The online version contains supplementary material available at 10.1007/s00436-025-08463-7.

## Introduction

*Trichomonas vaginalis*, which causes trichomoniasis, is the most common nonviral sexually transmitted disease in humans, with 276 million cases reported new cases each year (World Health Organization; 2012). *T. vaginalis* can cause long-term symptomatic infections of the vulvar and urethral regions of the genital tract (Petrin et al. 1998). Importantly, *T. vaginalis* infection is associated with an increased risk of HIV infection (McClelland et al. 2007), the development of prostate cancer (Sutcliffe et al. 2012; Twu et al. 2014), and complications during pregnancy, such as premature and low-weight births (Cotch et al. 1997).

The 2019 World Health Organization (WHO) report highlights the global epidemiological burden of trichomoniasis in 2016. The prevalence among women aged 15–49 years was estimated at 5.3% (95% uncertainty interval (UI): 4.0–7.2), significantly higher than the 0.6% prevalence observed in men of the same age group (95% UI: 0.4–0.9). The global incidence of new cases in 2016 was approximately 156 million (95% UI: 103.4–231.2 million), underlining the substantial worldwide impact of the infection. Regionally, the highest prevalence rates among women were recorded in the African region, reaching 11.7% (95% UI: 8.6–15.6), while lower rates were observed in other regions, such as the Americas, South-East Asia, and the Eastern Mediterranean, with estimates ranging from 2.5 to 7.7%. Among men, the prevalence was consistently lower across all regions, with the highest rates of 1.3% (95% UI: 0.7–1.8) also in the African Region (Rowley et al. 2019).

When analyzed by income classifications according to the World Bank, the burden of trichomoniasis demonstrated clear socioeconomic disparities. Low-income countries exhibited the highest prevalence, reflecting a disproportionate burden in resource-limited settings. In contrast, high-income countries reported the lowest prevalence. Upper-middle-income countries showed intermediate levels of prevalence, with a moderate burden compared to the extremes observed in low- and high-income countries. This stratification underscores the critical influence of socioeconomic factors in the global distribution and control of trichomoniasis (Rowley et al. 2019).

Currently, metronidazole, tinidazole, and secnidazole are used for treating *T. vaginalis* infections (de Brum Vieira et al. 2017; Muzny et al. 2022). The present recommended therapy for trichomoniasis, based on updated guidelines, prioritizes multidose oral metronidazole as the treatment of choice for women (Workowski 2021). The centers for disease control and prevention (CDC) recommend a regimen of 500 mg twice daily for 7 days, supported by randomized controlled trials demonstrating the superior efficacy of this approach compared to a single-dose regimen. For men, the standard treatment is a single 2-g dose of metronidazole, with an alternative option of a single 2-g dose of tinidazole for both sexes (Workowski 2021; Kissinger et al. 2018). Secnidazole, a next-generation 5-nitroimidazole, has been approved for trichomoniasis treatment in men, women, and adolescents aged 12 years or older. Administered as a single 2-g oral dose, secnidazole has shown comparable efficacy and safety to metronidazole and tinidazole (Muzny et al. 2021). However, it is not yet widely incorporated into standard treatment guidelines. In pregnant women, metronidazole remains the preferred therapy due to its well-established safety profile and lower cost (Workowski 2021). Conversely, tinidazole and secnidazole are contraindicated during pregnancy (Muzny et al. 2022). Additionally, breastfeeding is not recommended during treatment with these agents and should be avoided for several days afterward (Muzny and Kissinger 2023).

Despite their efficacy, clinical failure of metronidazole treatment has been observed since 1959 (Watt and Jennison 1960), with failure rates ranging from ∼4% in the USA (Kirkcaldy et al. 2012) to 17% in Papua New Guinea (Upcroft et al. 2009).

Resistance to metronidazole, the cornerstone of trichomoniasis treatment, presents a clinical challenge in managing *T. vaginalis* infections. The prevalence of metronidazole resistance has been reported to range from 2.2 to 9.6%, highlighting its relatively uncommon but significant occurrence (Graves et al. 2020; Kirkcaldy et al. 2012). Even with multidose metronidazole therapy, considered more effective than single-dose regimens, a breakthrough infection rate of 11% was observed in a randomized controlled trial, underscoring the persistence of resistant strains (Kissinger et al. 2018).

Tinidazole has demonstrated efficacy in treating metronidazole-resistant *T. vaginalis*. Cases of resistance often require prolonged therapy, such as 2 g of tinidazole administered orally daily for 7 days. For patients who fail this regimen, an alternative approach involving high-dose oral tinidazole (2–3 g daily in divided doses) combined with intravaginal tinidazole (500 mg twice daily for 14 days) has been suggested (Workowski 2021; Sobel et al. 2001).

There is limited evidence on the effectiveness of secnidazole in addressing metronidazole-resistant infections. However, a recent case study documented the successful resolution of metronidazole-resistant *T. vaginalis* using an extended 14-day course of secnidazole (2 g daily) in combination with intravaginal boric acid (600 mg twice daily). These findings highlight the need for further research to optimize treatment strategies for resistant infections (McNeil et al. 2023).

Metronidazole is a prodrug requiring reduction at its nitro group to generate toxic nitroradicals. This activation occurs in anaerobic conditions, making it relatively safe for aerobic organisms. The specific toxic agents include the 5-nitroradical anion and 5-nitrosoimidazole, which damage intracellular components (Leitsch 2019).

Resistance mechanisms are classified as in vitro (anaerobic) resistance, caused by loss of nitroreductase activity, and clinical (aerobic) resistance, linked to impaired oxygen scavenging (Leitsch 2019).

Often, infections caused by *T. vaginalis* that exhibit reduced susceptibility to 5-nitroimidazole drugs like metronidazole or tinidazole can still be cleared with higher-than-standard doses of these drugs. However, cases of “true resistance” involve strains with resistance mechanisms that render higher doses or alternative 5-nitroimidazole drugs ineffective. These situations pose significant treatment challenges due to the limited availability of alternative therapies and underscore the importance of developing novel strategies to address metronidazole-resistant *T. vaginalis* (Sobel et al. 2001).

In clinical strains resistant to metronidazole, resistance was only detected in the presence of oxygen, and these isolates were shown to have compromised oxygen scavenging mechanisms (Yarlett et al. 1986a, b), which likely results in elevated intracellular oxygen levels. A reduction in flavin reductase activity, exerted by flavin reductase 1 (FR1), was noted in both anaerobic and aerobic resistance types (Kulda 1999; Leitsch et al. 2009; 2012). Initially identified as an oxygen scavenger in *T. vaginalis* (Linstead and Bradley 1988), FR1 was later found to be the primary source of intracellular hydrogen peroxide (Chapman et al. 1999). Its role in removing oxygen was deemed essential for metronidazole resistance in both lab-induced and clinical strains (Leitsch et al. 2014).

However, recent findings challenge this view. A *T. vaginalis* cell line with early-stage resistance showed an 80% reduction in FR1 activity without affecting oxygen scavenging (Gehl et al. 2021), suggesting that FR1 reduction does not impair oxygen scavenging. This casts doubt on the current model of metronidazole resistance.

In our latest study (Mayr et al. 2024), we analyzed the total protein expression of metronidazole-susceptible and resistant strains to identify proteins specifically linked to resistance. Unexpectedly, the number of identified proteins was very low. When comparing the protein expression profiles of resistant clinical isolates with those of strains where resistance was induced in the lab, we discovered only one protein consistently downregulated across all datasets. This protein was FR1, and we discovered that it is able to reduce ferric iron within the cell. We further proposed a new mechanism for metronidazole activation that involves ferrous iron binding to proteins, making them susceptible to forming complexes with metronidazole. When these iron-protein-metronidazole complexes resolve, metronidazole radicals are generated, which rapidly react with nearby proteins, causing breaks in their peptide backbone. Therefore, in resistant *T. vaginalis*, FR1 is not expressed, protecting proteins from the effects of metronidazole (Mayr et al. 2024).

Comparative large-scale genetic and proteomics comparisons studies of metronidazole-sensitive and -resistant strains have highlighted the need to explore beyond candidate genes traditionally associated with resistance, such as PFOR, ferredoxin, and thioredoxin reductase, as alterations in these genes do not account for all cases of resistance (Leitsch et al. 2014; Bradic et al. 2017; Mayr et al. 2024). Nitroreductases (Ntrs) were also suggested among the enzymes responsible for the reduction of 5-nitroimidazoles (Pal et al. 2009). A study by Pal et al. showed that Ntr8 has nitroreductase activity and can reduce metronidazole (Pal et al. 2009). Paulish-Miller et al. also identified single-nucleotide polymorphisms (SNPs) in two nitroreductase genes (*ntr4* and *ntr6*) (Paulish-Miller et al. 2014). These SNPs, which introduced stop codons, were associated with metronidazole resistance. Isolates with either or both mutations showed greater metronidazole resistance (Paulish-Miller et al. 2014). Bradic et al. (2017) found that the B7268 strain had downregulated Ntr6, Ntr7, Ntr-like 1, Ntr-like 2, Ntr-like 3, oxidoreductase 1, 2, and 3, while other metronidazole-resistant strains had downregulated Ntr-like 3, oxidoreductase 1, 2, and 3. Among these, oxidoreductase 1 is particularly interesting because it belongs to the “old yellow enzyme” (OYE) family, which includes flavin enzymes of mostly unknown function. One member of this family, TcOYE, is a major drug-metabolizing enzyme in *Trypanosoma cruzi* (Bradic et al. 2017). Thus, oxidoreductase 1 is a strong candidate for a metronidazole-activating enzyme in *T. vaginalis*.

Therefore, our study is aimed at addressing some of the gaps and at identifying how factors such as nitroreductases, nitroreductase-like proteins, and oxidoreductases contribute to metronidazole resistance in *T. vaginalis*. Additionally, we aimed to determine if efflux pump inhibitors approved for medical use in humans can reverse metronidazole resistance as efflux pumps are involved in metronidazole resistance in at least some of the resistant isolates (Bradic et al. 2017).

## Material and methods

### *T. vaginalis* strains and culture

In this study, all *T. vaginalis* strains were cultivated in TYM medium (trypticase, yeast extract, maltose medium) as previously described (Diamond et al. 1957). The strains examined included the metronidazole-susceptible isolates C1 (ATCC 30001), G3 (PRA-98), and JH31A#4 (ATCC 30236). Additionally, CDC085 (ATCC 50143) and BRIS/92/STDL/B7268 were clinical isolates resistant to metronidazole. For simplicity, JH31A#4 and BRIS/92/STDL/B7268 are referred to as JH31A and B7268, respectively, in the text. A highly metronidazole-resistant cell line of C1 had been previously generated (Leitsch et al. 2009). Strain C1 was originally isolated in 1956 from the vaginal exudate of a woman with acute vaginitis. Strain G3 was isolated in 1973 from a clinical specimen in Beckenham, United Kingdom. JH31A was obtained from an endocervical swab at Johns Hopkins Hospital in Baltimore, Maryland, USA, in 1963. CDC085 was isolated during the study conducted between July 1982 and October 1983 from a patient attending the sexually transmitted disease clinic at the Columbus Health Department, Columbus, Ohio (Müller et al. 1988). Strain B7268, a metronidazole-resistant isolate, was identified in Brisbane, Australia, from a 54-year-old woman who had recurrent trichomoniasis for 18 months and failed multiple courses of metronidazole and tinidazole treatment (Voolmann and Boreham 1993). The C1, G3, JH31A, and CDC085 strains were purchased from LGC Standards, while strain B7268 was kindly provided by Melissa Conrad and Jane Carlton (New York University Langone Medical Center, USA).

### Minimal lethal concentration assay

Minimal lethal concentrations (MLCs) were assessed using 96-well plates as previously described by Mayr et al. (2024). In summary, tinidazole, zosuquidar, elacridar, pyrimethamine, and cimetidine were serially diluted in TYM medium to a final volume of 100 μL. Then, 100 μL of an inoculum containing 10,000 cells was added to each well. The plates were incubated at 37 °C for 48 h in airtight 2.5 L boxes under microaerophilic conditions (O_2_: 6.2–13.2%, CO_2_: 2.5–9.5%) maintained by CampyGen (Thermo Scientific). After incubation, MLCs were determined via light microscopy. The MLC was defined as the concentration at which cells were no longer motile, indicating cell death. Each experiment was conducted three times, with technical duplicates to ensure reproducibility.

### Checkerboard assay

Assays were conducted in 96-well plates using combinations of metronidazole/tinidazole with zosuquidar, elacridar, pyrimethamine, or cimetidine as outlined by Bellio et al. (2021). Serial dilutions of metronidazole or tinidazole were performed horizontally across rows A–G, while serial dilutions of zosuquidar, elacridar, pyrimethamine, or cimetidine were conducted vertically across columns 2–11, creating 77 unique combinations of test compounds (plate layout shown in supplementary Fig. 1). Row H contained only serial dilutions of metronidazole/tinidazole, and column 1 contained only serial dilutions of the other drugs. After dilutions, each well was inoculated with 10,000 cells and incubated for 48 h under microaerophilic conditions (O_2_: 6.2–13.2%, CO_2_: 2.5–9.5%) using CampyGen (Thermo Scientific). Cell viability was then assessed microscopically, and MLC values were recorded for each drug both individually and in combination. The fractional inhibitory concentration index (FICI) was calculated to determine drug interactions using the formula: FICI = (MIC_AB_/MIC_A_) + (MIC_BA_/MIC_B_), where MIC_AB_ is the minimum inhibitory concentration (MIC) of drug A in combination, MICA is the MIC of drug A alone, MIC_BA_ is the MIC of drug B in combination, and MIC_B_ is the MIC of drug B alone. Drug combination effects were classified as synergistic (∑FICI ≤ 0.5), additive (∑FICI between 0.5 and 1.0), no effect (∑FICI between 1.0 and 4.0), and antagonistic (∑FICI ≥ 4.0).

### RNA isolation and cDNA synthesis

Total cellular RNA was isolated using the innuPREP RNA Mini Kit (Analytik Jena) following the manufacturer’s instructions. Before cDNA synthesis, RNA was treated with RNA-free DNase I (1 U per µg of total RNA) (Thermo Scientific) to remove any residual DNA. First-strand cDNA synthesis was then carried out using the RevertAid First Strand cDNA Synthesis Kit (Thermo Scientific) according to the manufacturer’s instructions. In brief, total RNA was mixed with random hexamer primers and nuclease-free water to a final volume of 12 µL and incubated at 65 °C for 5 min. Each reaction was then supplemented with RiboLock RNase Inhibitor (20 U/µL), 10 mM dNTP mix, RevertAid M-MuLV RT (200 U/µL), and 5X reaction buffer to a total volume of 20 µL, followed by a 5-min incubation at 25 °C and then 60 min at 42 °C. Reactions were terminated by heating at 70 °C for 5 min. The resulting cDNA was used directly for RT-PCR applications or stored at − 20 °C for up to 1 week.

### RT-PCR and electrophoresis

RT-PCR amplification was conducted using the HotStarTaq Master Mix Kit (QIAGEN). Each reaction, with a total volume of 50 µL, was prepared in DNA, DNase, and RNase-free 0.2 mL PCR soft tubes (Biozym). The reaction mix included 2 µL of cDNA template, 2X HotStarTaq Master Mix, 2 µM of each primer (listed in Supplementary Table 1), and RNase-free water. The tubes were placed in a UNO96 Thermocycler (VWR) with the following cycling program: initial heat activation at 95 °C for 15 min, denaturation at 94 °C for 30 s, annealing at 55 °C or 60 °C (for nitroreductase 7, nitroreductase 11, nitroreductase-like 3, and oxidoreductase 2) for 30 s, and extension for 50 s (for products smaller than 1000 bp) or 1 min 30 s (for products larger than 1000 bp). The program included 34 cycles and finished with a final extension at 72 °C for 10 min.

For electrophoresis, a 1% agarose gel in 1 × TBS with 0.5 µg/mL ethidium bromide was prepared. RT-PCR products (5 µL) were mixed with 6X Purple Loading Dye (New England BioLabs) and loaded onto the gel along with 5 µL of GeneRuler 1 kb DNA Ladder (Thermo Scientific). Electrophoresis was performed in a Wide Mini-Sub Cell GT Cell horizontal system (Bio-Rad) for 25 min at a constant voltage of 110 V, provided by a PowerPac Power Supply (Bio-Rad). Results were documented using a gel documentation system (PeQLab Biotechnologie GmbH).

### Recombinant protein expression of hexahistidine-tagged nitroreductases, nitroreductase-like proteins, and oxidoreductases in *E. coli*

Genomic DNA from the *T. vaginalis* G3 strain was used as a template to amplify 11 nitroreductase genes (Ntr1-Ntr11), 3 nitroreductase-like protein genes (Ntr-like 1–3), and 3 oxidoreductase genes (OxR 1–3). The sequences of the primers used for amplification are provided in Supplementary Table 1. The forward primers were designed to include an NdeI restriction site, while the reverse primers contained an XhoI restriction site along with a hexahistidine tag for simplified protein purification. However, the native sequences of the Ntr7, Ntr11, and Ntr-like 3 genes already contained an internal NdeI restriction site, which would have interfered with downstream cloning steps. To resolve this, we introduced synonymous nucleotide substitutions at these internal sites, thereby preserving the amino acid sequence while eliminating the unwanted NdeI sites. The modified sequences were synthesized by Eurofins Genomics. The detailed sequences of these genes, including the synonymous nucleotide changes, are provided in Supplementary File 1. The PCR fragments and synthetized modified genes were ligated into pET17b vectors, and the resulting plasmids were propagated in 10-beta cells as per the manufacturer’s instructions. These plasmids were then used to transform BL21-AI cells, BL21 Codon Plus (for Ntr4 and Ntr6), SHuffle T7 Competent *E. coli* (for Ntr1), or the Origami strain (for OxR1). Transformed cells were selected on Luria–Bertani agar plates containing 100 mg/mL ampicillin. Recombinant protein expression was induced in BL21-AI with 0.2% L-arabinose and in BL21 Codon Plus with 0.4 mM Isopropyl β-D-1-thiogalactopyranoside (IPTG) and was carried out for 3 h at 37 °C. SHuffle T7 Competent *E. coli* and the Origami strain were induced with 0.1 mM IPTG overnight at 16 °C. Harvested cells were ground in a mortar and the proteins were purified using Ni–NTA spin columns (QIAGEN). The recombinant *T. vaginalis* nitroreductases were designated as recTvNtr1-11, the nitroreductase-like proteins as recTvNtr-like1-3, and the oxidoreductases as recTvOxR1-3.

### Nitroreductase activity assay

Nitroreductase activities were assessed following established protocols (Leitsch et al. 2009; Paunkov et al. 2022). Due to the strong absorption of metronidazole at 340 nm, which overlaps with the wavelength used to measure NADPH and NADH oxidation, a cytochrome c-based assay was chosen for measuring metronidazole reduction (Leitsch et al. 2009; Paunkov et al. 2022). The reduction of metronidazole-mediated cytochrome c was monitored at 550 nm (Δε550 = 20 mM^−1^ cm^−1^). Reduction of the nitro group by Ntrs, Ntr-like proteins, and OxRs results in the reduction of one molecule of cytochrome c. The reaction buffer contained 100 mM Tris (pH 7.5), 0.2 mM NADPH, 50 µM cytochrome c, 1 mM metronidazole, and 5 µg/mL recTvNtr1-11, recTvNtr-like 1–3, or recTvOxR 1–3. For other nitro drugs, such as the nitrofuran furazolidone (20 µM), absorption at 340 nm (Δε340 = 6.2 mM^−1^ cm^−1^) was used to determine NADPH oxidation. The reaction buffer consisted of 100 mM Tris (pH 7.5), 0.2 mM NADPH, and 5 µg/mL recTvNtr1-11, recTvNtr-like 1–3, or recTvOxR 1–3. Spectrometric measurements were performed using a PerkinElmer Lambda 25 spectrometer. As a positive control, recombinant thioredoxin reductase (recTvTrxR), as previously described, was included (Leitsch et al. 2009).

### Ferric iron reduction assay

The ferric iron reductase activity of recTvNtrs, recTvNtr-like proteins, and recTvOxRs was measured as outlined by Mayr et al. (2024). A calibration curve was first established by measuring the absorption of bipyridyl-iron complexes at 522 nm, formed by reacting bipyridyl (400 µM) with known concentrations of ferrous iron sulfate (Fe_2_SO_4_) in 100 mM potassium phosphate buffer at pH 6.75. Absorption was recorded for 0, 10, 20, 30, 40, 50, and 60 µM Fe_2_SO_4_, with four independent measurements at each concentration. The reduction of 50 µM ferric iron chloride (FeCl_3_) in the same buffer was then assessed in the presence of 10 µg/mL recTvNtrs, recTvNtr-like proteins, or recTvOxRs, along with 20 µM FMN and 2 mM NADPH. The freshly formed Fe^2+^, resulting from the reduction of Fe^3+^ by these enzymes, was bound by bipyridyl, and the increase in absorption at 522 nm was recorded. The recorded values were plotted against the calibration curve, and RStudio and the ggplot2 package were used to create detailed graphs and visualizations (RStudio Team 2024; Wickham 2016).

## Results

### Susceptibility of *T. vaginalis* strains to metronidazole, tinidazole, and efflux pump inhibitors

The MLC assay demonstrated significant variation in the susceptibilities of *T. vaginalis* strains to metronidazole and tinidazole. Metronidazole-sensitive strains (C1 and G3) exhibited significantly lower MLCs compared to metronidazole-resistant strains (B7268 and CDC085). These differences underscore the impact of resistance mechanisms on drug efficacy. Tinidazole displayed either comparable or lower MLCs across all strains, suggesting its potential utility in overcoming resistance. Notably, the *p*-values from paired *t*-tests comparing metronidazole and tinidazole for each strain reveal significant differences for resistant strains (B7268: *p* = 0.008, CDC085: *p* = 0.013), while no significant differences were observed for sensitive strains (C1: *p* = 0.519, G3: *p* = 0.251). These findings highlight tinidazole’s promise, particularly against metronidazole-resistant strains (Table 1).

**Table 1:** MLCs of metronidazole and tinidazole for various *T. vaginalis* strains. Data are presented as mean ± standard deviation from three independent experiments. Statistical significance of differences between MLCs of metronidazole and tinidazole for each strain was determined using paired *t*-tests, with *p*-values provided

Strain	Metronidazole (µM)	Tinidazole (µM)	*p-*value
C1	6.25 ± 2.71	7.81 ± 2.71	0.519
G3	6.25 ± 2.71	3.91 ± 1.35	0.251
B7268	1000.00 ± 346.41	31.25 ± 10.83	0.008
CDC085	2000.00 ± 692.82	300.00 ± 0.00	0.013

In contrast, zosuquidar, elacridar, and cimetidine demonstrated no lethal effects even at concentrations up to 100 µM, implying their limited efficacy against both sensitive and resistant strains of *T. vaginalis*. Pyrimethamine, however, maintained a consistent MLC of 50 µM across all strains, suggesting it could potentially serve as an alternative to metronidazole (Table 2). However, it is important to note that while pyrimethamine showed some activity, it is not a stronger alternative to metronidazole compared to tinidazole.

**Table 2:** MLCs of zosuquidar, elacridar, pyrimethamine, and cimetidine in *T. vaginalis* strains. Measurements were performed in one biological replicate

Strain	Zosuquidar	Elacridar	Pyrimethamine	Cimetidine
C1	> 100 µM	> 100 µM	50 µM	> 100 µM
G3	> 100 µM	> 100 µM	50 µM	> 100 µM
B7268	> 100 µM	> 100 µM	50 µM	> 100 µM
CDC085	> 100 µM	> 100 µM	50 µM	> 100 µM

### Limited interaction and inhibitory effect of zosuquidar, elacridar, pyrimethamine, and cimetidine with metronidazole or tinidazole on *T. vaginalis* strains

The checkerboard assay was designed to assess the interactions between metronidazole or tinidazole and four other compounds (zosuquidar, elacridar, pyrimethamine, or cimetidine) against different strains of *T. vaginalis*. The interactions were evaluated using the FICI, which classifies the interactions as synergistic, additive, indifferent (no effect), or antagonistic. A FICI value between 1.0 and 4.0 indicates no significant interaction (no effect) between the drugs.

All combinations of metronidazole and tinidazole with zosuquidar, elacridar, and cimetidine yielded an FICI of 3, indicating no effect for these combinations (Tables 3 and 4). The combination of metronidazole and tinidazole with pyrimethamine yielded an FICI of 2.5, also indicating no effect.

**Table 3:** Fractional inhibitory concentration index of metronidazole and zosuquidar, elacridar, pyrimethamine, or cimetidine

Strain	FICI			
	Metronidazole + zosuquidar	Metronidazole + elacridar	Metronidazole + pyrimethamine	Metronidazole + cimetidine
**C1**	3	3	2.5	3
**G3**	3	3	2.5	3
**B7268**	3	3	2.5	3
**CDC085**	3	3	2.5	3

**Table 4:** Fractional inhibitory concentration index of tinidazole and zosuquidar, elacridar, pyrimethamine, or cimetidine

Strain	FICI			
	Tinidazole + zosuquidar	Tinidazole + elacridar	Tinidazole + pyrimethamine	Tinidazole + cimetidine
**C1**	3	3	2.5	3
**G3**	3	3	2.5	3
**B7268**	3	3	2.5	3
**CDC085**	3	3	2.5	3

Zosuquidar, elacridar, and cimetidine when combined with metronidazole or tinidazole do not exhibit synergistic or additive effects against any of the *T. vaginalis* strains tested. These combinations do not enhance the efficacy of either drug. Similarly, pyrimethamine does not show synergistic or additive effects when combined with metronidazole or tinidazole. Although it has some activity as noted in previous MLC assays, it does not improve the activity of these nitroimidazole drugs in combination.

The FICI values are consistent across all strains (C1, G3, B7268, CDC085), indicating that the lack of interaction is independent of the strain’s sensitivity or resistance to metronidazole. These results suggest that the resistance mechanisms of *T. vaginalis* are complex and may not be overcome by a single efflux pump inhibitor. It is likely that the combination of inhibitors targeting multiple resistance pathways would be more effective.

### Metronidazole-sensitive and metronidazole-resistant strains of *T. vaginalis* transcribe nitroreductases, nitroreductase-like proteins, and oxidoreductases

**Fig. 1:**
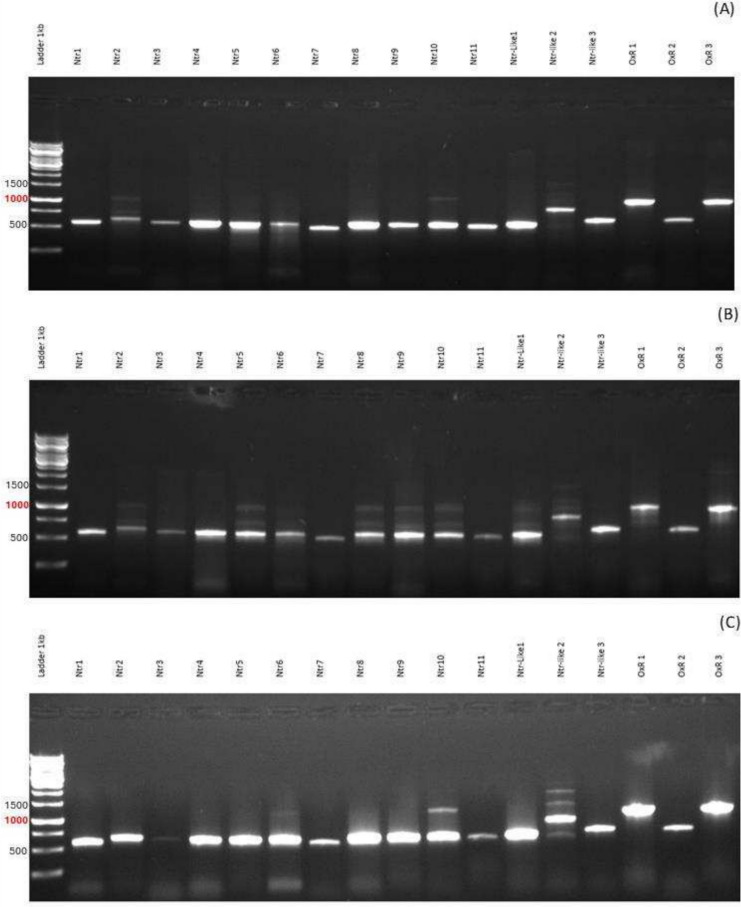
Agarose gel electrophoresis (1%) of RT-PCR products of Ntrs, Ntr-like proteins, and OxRs from metronidazole-sensitive strains C1 (**A**), G3 (**B**), and JH31A (**C**)

**Fig. 2:**
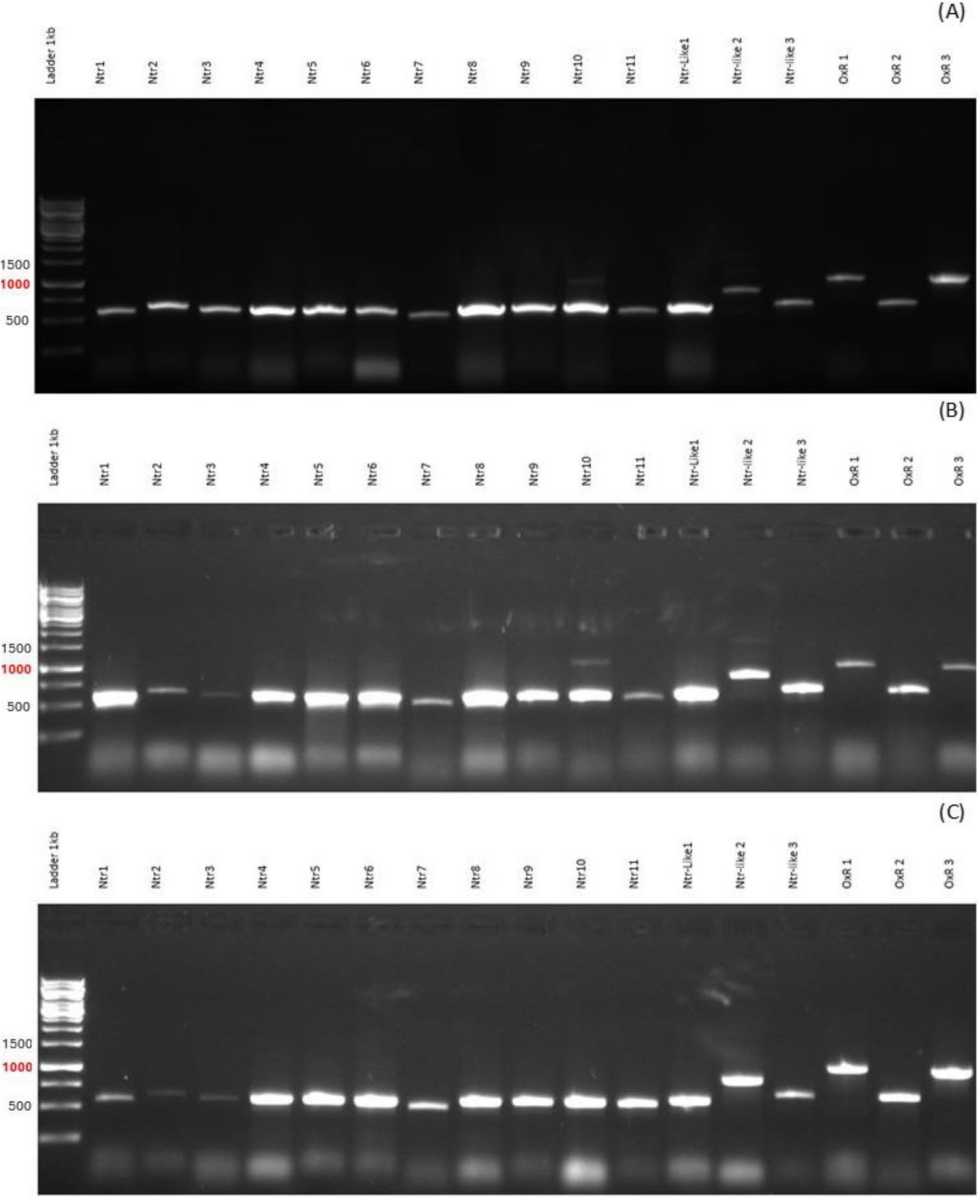
Agarose gel electrophoresis (1%) of RT-PCR products of Ntrs, Ntr-like proteins, and OxRs from metronidazole-resistant strains B7269 (**A**), CDC085 (**B**), and highly metronidazole-resistant C1 strain (**C**)

We aimed to confirm the expression of Ntrs, Ntr-like proteins, and OxRs in various metronidazole-sensitive and metronidazole-resistant strains of *T. vaginalis* including C1, G3, JH31A, B7268, CDC085, and the highly metronidazole-resistant C1 strain. The agarose gel electrophoresis results demonstrated the presence of RT-PCR products corresponding to Ntrs, Ntr-like proteins, and OxRs in all tested strains. This indicates that these strains transcribe the genes for these enzymes regardless of the level of metronidazole resistance (Figs. 1 and 2).

### Recombinant protein expression of hexahistidine-tagged nitroreductases, nitroreductase-like proteins, and oxidoreductases in *E. coli*

Among the proteins expressed, only recTvNtr8, recTvNtr9, and recTvNtr10 were successfully isolated from the BL21-AI strain. Other proteins could not be isolated using this approach due to insolubility, as the recombinant proteins were trapped in inclusion bodies. Attempts to use Origami and SHuffle strains, which are designed for the expression of redox-sensitive proteins, with other proteins were only successful for recTvNtr1 and recTvOxR1 when the temperature and inducer concentration were reduced.

For recTvNtr4 and recTvNtr6, the BL21 Codon Plus strain was employed, which is suitable for expressing potentially toxic gene products that might otherwise be sequestered in inclusion bodies to protect the cells. This approach was successful, yielding purified proteins following affinity purification on nickel columns.

The provided SDS-PAGE figure in the supplementary data shows the purified recombinant proteins (Supplementary Fig. 2).

### Lack of nitroreductase activity in recombinant nitroreductases and oxidoreductase 1

Our assay results indicated that none of the tested recombinant nitroreductases (recTvNtr1, recTvNtr4, recTvNtr6, recTvNtr8, recTvNtr9, recTvNtr10) and oxidoreductase (recTvOxR1) were able to reduce metronidazole. Further testing with the nitrofuran drug furazolidone, which has a higher midpoint potential and should theoretically be more readily reduced, also showed no reduction activity by any of the tested recombinant proteins. In both cases, only the positive control, recombinant thioredoxin reductase (recTvTrxR) (Leitsch et al. 2009), demonstrated nitroreductase activity. Proteins that were not isolated due to insolubility were not included in these assays.

### Ferric iron reductase activity observed in recTvNtr8

**Fig. 3:**
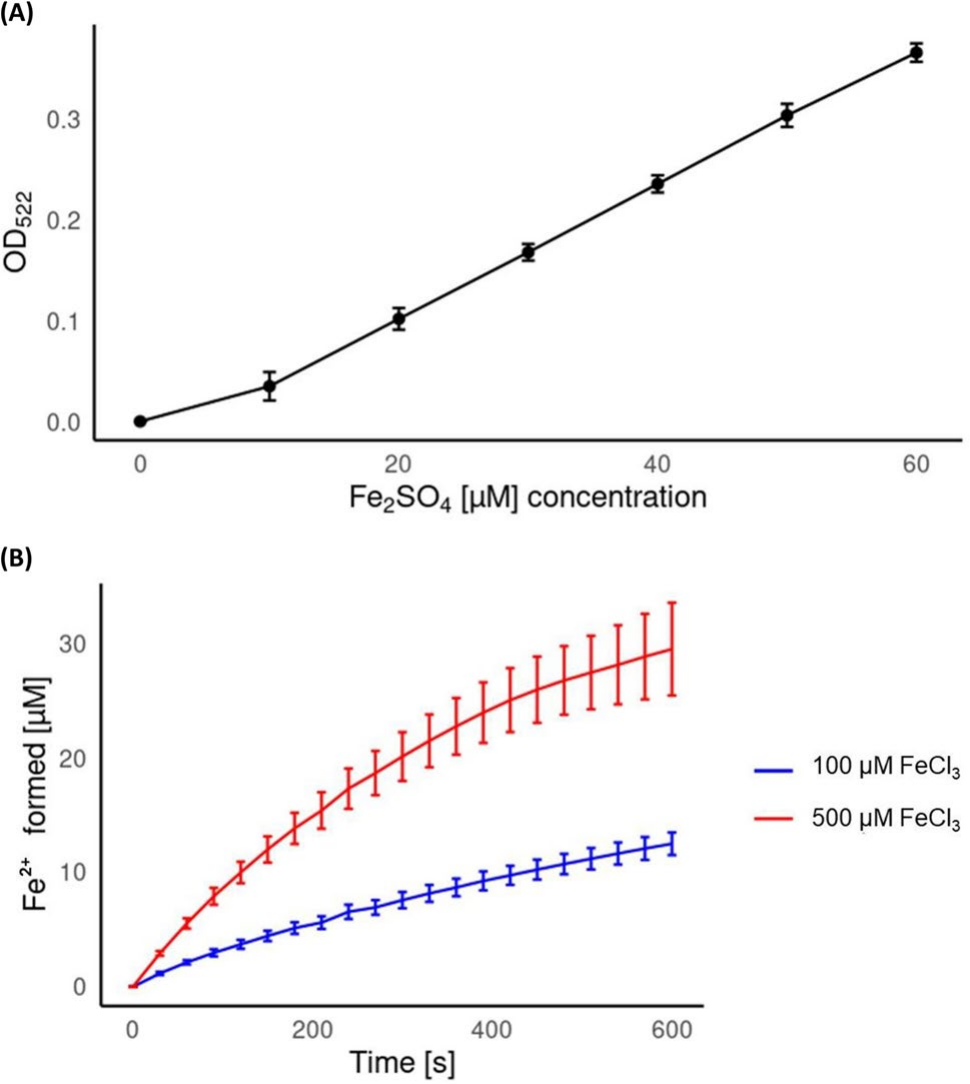
Calibration curve of known concentrations of Fe_2_SO_4_ (0 – 60 µM) in presence of bipyridyl measured at OD_522_ (**A**). Ferric iron reduction activity of recTvNtr8 determined through the quantification of complex formation of Fe^2+^ with bipyridyl (**B**). The absorption values from measurements obtained from recombinant protein and 100 or 500 µM FeCl_3_ were matched against the calibration curve and presented as average values with error bars indicating standard deviation (SD ±). All measurements were performed in triplicates

The ferric iron reductase activity assay results showed that among the tested recombinant proteins, only recTvNtr8 exhibited detectable ferric iron reductase activity. This enzyme was able to reduce ferric iron, as indicated by the formation of bipyridyl-iron complexes at 522 nm (Fig. 3). The rate of complex formation was dependent on and limited by the concentration of FeCl_3_. Without recTvNtr8, bipyridyl-Fe^2+^ complexes did not form. All tested strains transcribe the genes for nitroreductases regardless of their metronidazole resistance levels (Figs. 1 and 2). While our assay confirmed the iron-reducing activity of purified recTvNtr8, we did not evaluate its activity in cell extracts from sensitive or resistant strains due to potential ambiguity introduced by other enzymes contributing to iron-reducing activity in such extracts. We acknowledge that testing Ntr8 activity in this context would provide valuable insights into its role in resistance mechanisms, and we consider this an important direction for future research.

## Discussion

Trichomoniasis significantly impacts individuals and communities, with many cases remaining asymptomatic and undiagnosed due to the low sensitivity of the commonly used diagnostic method, the wet mount. The public health implications of trichomoniasis have prompted efforts to enhance intervention effectiveness. Current treatments rely almost exclusively on 5-nitroimidazoles, raising concerns about potential widespread drug resistance and the variability in effectiveness among different 5-nitroimidazoles against *T. vaginalis*.

Unlike many bacterial resistance mechanisms, resistance to 5-nitroimidazoles in *Trichomonas* appears relative rather than absolute. The rapid emergence of metronidazole resistance suggests pre-existing mechanisms of drug tolerance (Robinson 1962; Cudmore et al. 2004; Jenks and Edwards 2002; Kouhsari et al. 2019). For example, increasing the dosage of metronidazole can often treat infections that do not respond to the standard dose, indicating that the enzymes activating metronidazole are essential for vital cellular functions and that complete loss of these enzymes would be lethal to the parasite (Lossick et al. 1986). Additionally, the complexity of interactions within the vaginal environment, such as drug levels, intra-vaginal redox potential, and microbiota composition, influences drug resistance levels (Lossick et al. 1986). However, high-dose metronidazole treatment is limited by patient tolerance due to side effects such as nausea and peripheral neuropathy, necessitating the careful consideration of risks and benefits (Lossick et al. 1986; Workowski et al. 2015).

We aimed to evaluate the effects of metronidazole and tinidazole on different metronidazole-resistant strains. Tinidazole, like metronidazole, is a 5-nitroimidazole that is effective against anaerobic bacteria and protozoa (Sawyer et al. 1996; Bergan 1985). Tinidazole has a plasma elimination half-life that is about twice as long as metronidazole’s (12–14 h vs. 6–7 h) (Sutton et al. 1999; Sawyer et al. 1996). In clinical practice, tinidazole has shown to be equally effective as metronidazole in treating trichomoniasis when given at the same dose. Laboratory studies also indicate that tinidazole typically requires a lower minimal lethal concentration than metronidazole (Sawyer et al. 1996). Additionally, some *Trichomonas* strains from patients who do not respond to metronidazole are more susceptible to tinidazole. The side effects of tinidazole are similar to those of metronidazole, but several reports suggest they occur less frequently and are less severe (Roe 1985). There are reported cases of high-dose tinidazole successfully treating vaginal trichomoniasis that was resistant to high doses of metronidazole Lossick and Kent 1991; Gillette et al. 1999; Voolmann and Boreham 1993; Hamed and Studemeister 1992; Saurina et al. 1998). Although some studies have found significantly lower minimal lethal concentration for tinidazole against aerobic organisms compared to metronidazole, this difference was not always apparent, even in patients who were cured with tinidazole (Saurina et al. 1998). In our study, tinidazole demonstrated better effectiveness against metronidazole-resistant strains, consistent with other studies showing lower minimal lethal concentration levels for tinidazole compared to metronidazole. Despite increased resistance to metronidazole correlating with decreased sensitivity to tinidazole, tinidazole remains a viable treatment alternative.

Our research also looked at whether combining metronidazole or tinidazole with other drugs could enhance their effectiveness against resistant strains and whether efflux pump inhibitors could reverse metronidazole resistance. *T. vaginalis* has many multidrug resistance (MDR) genes that encode efflux pumps, including over 90 ATP-binding cassette (ABC) transporters and 48 homologs from the multi-antimicrobial extrusion (MATE) family. Some of these genes, like CAA53758 and TVAG_254060, are more active in metronidazole-resistant cell lines (Dunne et al. 2003). Johnson et al. (1994) studied seven clinical isolates of *T. vaginalis* that were 5–10 times less sensitive to metronidazole than drug-sensitive strains. Their findings showed variations in the expression and number of Tvpgp genes in resistant strains. Three of the seven resistant strains overexpressed Tvpgp by 2–20 times, and four of these strains lacked one of the two genes found in drug-sensitive strains (Johnson et al. 1994).

Bradic et al. (2017) sequenced multiple clinical isolates and lab-derived lines to identify genetic markers and mechanisms of metronidazole resistance. They found that resistant strains commonly showed changes in the expression of genes related to drug activation (e.g., FR1), detoxification (e.g., nitroreductase), and accumulation (e.g., multidrug resistance pump). Both laboratory-derived and clinical isolates had increased expression of one ABC transporter (TVAG_254060) and two multidrug resistance pumps (TVAG_291970 and TVAG_210540) (Bradic et al. 2017).

Inhibiting these efflux pumps with MDR modulators (zosuquidar and elacridar) and MATE inhibitors (cimetidine and pyrimethamine) was hypothesized to restore metronidazole sensitivity. Zosuquidar and elacridar, initially developed as inhibitors of ATP-binding cassette (ABC) transporters such as P-glycoprotein (ABCB1), have shown significant potential for repurposing in the treatment of leishmaniasis and *Fusobacterium nucleatum* (Pérez-Victoria et al. 2006; Hodgkinson and Sharples (2002). These agents are primarily used to enhance the efficacy of chemotherapeutic drugs in overcoming MDR in cancer. Zosuquidar, for instance, has been employed to inhibit ABCB1-mediated drug efflux and improve the intracellular accumulation of chemotherapeutics like daunorubicin (Tang et al. 2008). Similarly, elacridar demonstrates activity against both ABCB1 and ABCG2 transporters, rendering cancer cells more susceptible to various anticancer agents (Shukla et al. 2011). Zosuquidar and elacridar inhibit the Leishmania-specific ABC transporter LtrMDR1, a key mediator of resistance to miltefosine, the first-line treatment for leishmaniasis. By blocking drug efflux, they restore miltefosine’s efficacy in resistant strains, highlighting their potential as adjunctive therapies for leishmaniasis alongside their established use in oncology (Pérez-Victoria et al. 2006). Pyrimethamine, an antiparasitic drug used to treat malaria and toxoplasmosis, inhibits dihydrofolate reductase (DHFR), disrupting nucleotide synthesis essential for parasite replication. While minimally affecting the human enzyme, it targets protozoan parasites effectively (Kabra et al. 2019; Ben-Harari et al. 2017). Recently, pyrimethamine has been explored for leishmaniasis treatment due to the parasite’s reliance on the folate biosynthesis pathway (Kabra et al. 2019). Although its exact mechanism in Leishmania is under study, its known action against other protozoa and potential for combination therapies make it a promising candidate for repurposing in leishmaniasis. Cimetidine, a histamine-2 (H2) receptor antagonist, is primarily used to treat acid-related stomach conditions like gastroesophageal reflux disease and peptic ulcers by reducing acid secretion. It also has immunomodulatory effects, promoting a T-helper 1 (TH1) response and suppressing T-helper 2 (TH2) activity. In Lyme disease, caused by *Borrelia burgdorferi*, which can suppress TH1 responses to persist in the host, cimetidine may enhance TH1-associated cytokines (IL-12, TNF-α, IFN-γ) and suppress TH2 cytokines (IL-10), restoring immune balance (Embers et al. 2004). Early cimetidine therapy could improve antibiotic efficacy and reduce pathogen persistence, enhancing clinical outcomes (Shemenski 2019).

With this background on the mechanisms and efficacy of these drugs against other microorganisms, we decided to evaluate their potential alone and in combination with metronidazole and tinidazole, hypothesizing that they might similarly enhance the efficacy of these 5-nitroimidazoles against *T. vaginalis*. Using a checkerboard assay, we tested various combinations, but the tested efflux pump inhibitors did not significantly enhance the efficacy of metronidazole or tinidazole. Although pyrimethamine showed some activity, it did not improve the efficacy of the 5-nitroimidazoles in combination.

We further hypothesized that several Ntrs encoded in the *T. vaginalis* genome contribute to metronidazole activation and are less expressed or less active in resistant strains. However, our tests could not confirm this activity in any of the enzymes examined. Interestingly, our previous proteomics study (Mayr et al. 2024) did not detect all 11 Ntrs, 3 Ntr-like proteins, and 3 OxRs. Our proteomic analysis failed to detect Ntr1, Ntr3, Ntr7, Ntr9, and OxR2, with no clear trend suggesting Ntrs are downregulated in resistant strains. The insolubility of some enzymes, as we encountered it during isolation of recombinant proteins, might explain why they were not detected in the proteomics analysis, indicating a limitation of the approach. Pal et al. (2009) also noted that Ntrs mRNAs were variably expressed by cultured *Trichomonas* isolates, with no relationship to metronidazole sensitivity. Our RT-PCR results showed that the tested strains expressed all these genes, with variations in their expression but no clear downregulation trend in resistant strains.

Given the inability to confirm nitroreductase activity in resistant strains, we hypothesized that these enzymes might compensate for FR1 function by reducing ferric iron. Our findings confirm that recTvNtr8 can reduce ferric iron, though less effectively than FR1. This activity may influence iron availability, which is essential for metabolic processes and gene regulation in *T. vaginalis* (Argaez-Correa et al. 2019). Iron supplementation during metronidazole treatment has been shown to lower MLCs in sensitive strains under both aerobic and anaerobic conditions (Elwakil et al. 2017). These results suggest that Ntr8’s ability to reduce iron could play a role in resistance by modulating intracellular iron levels. However, further studies are needed to directly link this activity to resistance mechanisms and to determine whether Ntr8’s role is compensatory or complementary to other resistance mechanisms.

## Conclusion

Our findings indicate that efflux pump inhibitors do not significantly enhance the efficacy of either metronidazole or tinidazole. Consistent with previous studies, tinidazole demonstrated greater effectiveness against metronidazole-resistant strains, reaffirming its potential as a superior alternative in such cases. The role of nitroreductases and other enzymes in metronidazole resistance remains unclear, highlighting the need for further research to elucidate these mechanisms. A deeper understanding of resistance pathways and the development of new treatment strategies are essential for improving the management of trichomoniasis and reducing its public health burden.

## Supplementary Information

Below is the link to the electronic supplementary material.

Supplementary file1 (DOCX 127 KB)

Supplementary file2 (DOCX 57 KB)

Supplementary file3 (DOCX 14 KB)

Supplementary file4 (DOCX 20 KB)

## Data Availability

No datasets were generated or analysed during the current study.

## References

[CR1] Argaez-Correa W, Alvarez-Sanchez ME, Arana-Argaez VE, Ramirez-Camacho MA, Novelo-Castilla JS, Coral-Martinez TI, Torres-Romero JC (2019) The role of iron status in the early progression of metronidazole resistance in *Trichomonas vaginalis* under microaerophilic conditions. J Eukaryot Microbiol 66:309–315. 10.1111/jeu.12671 30047563 10.1111/jeu.12671

[CR2] Bellio P, Fagnani L, Nazzicone L, Celenza G (2021) New and simplified method for drug combination studies by checkerboard assay. MethodsX 8:101543. 10.1016/j.mex.2021.101543 34754811 10.1016/j.mex.2021.101543 PMC8563647

[CR3] Ben-Harari RR, Goodwin E, Casoy J (2017) Adverse event profile of pyrimethamine-based therapy in toxoplasmosis: a systematic review. Drugs r&d 17:523–544. 10.1007/s40268-017-0206-8 10.1007/s40268-017-0206-8 PMC5694419 28879584

[CR4] Bergan T (1985) Antibacterial activity and pharmacokinetics of nitroimidazoles: a review. Scand J Infect Dis Suppl 46:64–71 3865352

[CR5] Bradic M, Warring SD, Tooley GE, Scheid P, Secor WE, Land KM, Huang PJ, Chen TW, Lee CC, Tang P, Sullivan SA, Carlton JM (2017) Genetic indicators of drug resistance in the highly repetitive genome of *Trichomonas vaginalis*. Genome Biol Evol 9:1658–1672. 10.1093/gbe/evx110 28633446 10.1093/gbe/evx110 PMC5522705

[CR6] Chapman A, Linstead DJ, Lloyd D (1999) Hydrogen peroxide is a product of oxygen consumption by *Trichomonas vaginalis*. J Biosci 24:339–344. 10.1007/BF02941248

[CR7] Cotch MF, Pastorek JG II, Nugent RP, Hillier SL, Gibbs RS, Martin DH, Eschenbach DA, Edelman R, Carey CJ, Regan JA, Krohn MA (1997) *Trichomonas vaginalis* associated with low birth weight and preterm delivery. Sex Transm Dis 24:353–360. 10.1097/00007435-199707000-00008 9243743 10.1097/00007435-199707000-00008

[CR8] Cudmore SL, Delgaty KL, Hayward-McClelland SF, Petrin DP, Garber GE (2004) Treatment of infections caused by metronidazole-resistant *Trichomonas vaginalis*. Clin Microbiol Rev 17:783–793. 10.1128/cmr.17.4.783-793.2004 15489348 10.1128/CMR.17.4.783-793.2004 PMC523556

[CR9] de Brum VP, Tasca T, Secor WE (2017) Challenges and persistent questions in the treatment of trichomoniasis. Curr Top Med Chem 17:1249–1265. 10.2174/1568026616666160930150429 27697044 10.2174/1568026616666160930150429 PMC10169758

[CR10] Diamond LS (1957) The establishment of various trichomonads of animals and man in axenic cultures. J Parasitol 3:488–490. 10.2307/3274682 13463700

[CR11] Dunne RL, Dunn LA, Upcroft P, O’Donoghue PJ, Upcroft JA (2003) Drug resistance in the sexually transmitted protozoan *Trichomonas vaginalis*. Cell Res 13:239–249. 10.1038/sj.cr.7290169 12974614 10.1038/sj.cr.7290169

[CR12] Elwakil HS, Tawfik RA, Alam-Eldin YH, Nassar DA (2017) The effect of iron on metronidazole activity against *Trichomonas vaginalis* in vitro. Exp Parasitol 182:34–36. 10.1016/j.exppara.2017.09.021 28935536 10.1016/j.exppara.2017.09.021

[CR13] Embers ME, Ramamoorthy R, Philipp MT (2004) Survival strategies of *Borrelia burgdorferi*, the etiologic agent of Lyme disease. Microbes Infect 6:312–318. 10.1016/j.micinf.2003.11.014 15065567 10.1016/j.micinf.2003.11.014

[CR14] Gehl V, Paunkov A, Leitsch D (2021) A reassessment of the role of oxygen scavenging enzymes in the emergence of metronidazole resistance in trichomonads. Int J Parasitol Drugs Drug Resist 16:38–44. 10.1016/j.ijpddr.2021.04.004 33962363 10.1016/j.ijpddr.2021.04.004 PMC8113990

[CR15] Gillette H, Schmid GP, Moswe D (1999) Metronidazole-resistant *Trichomonas vaginalis*, a case series, 1985–1998 [abstract 067], XIIIth meeting of the International Society of Sexually Transmitted Disease Research (Denver), 1999 July 11–14.

[CR16] Graves KJ, Novak J, Secor WE, Kissinger PJ, Schwebke JR, Muzny CA (2020) A systematic review of the literature on mechanisms of 5-nitroimidazole resistance in *Trichomonas vaginalis*. Parasitology 147(13):1383–1391. 10.1017/S0031182020001237 32729451 10.1017/S0031182020001237 PMC7677174

[CR17] Hamed KA, Studemeister AE (1992) Successful response of metronidazole-resistant trichomonal vaginitis to tinidazole. Sex Transm Dis 19:339–340 1492261

[CR18] Hodgkinson R, Sharples D (2002) Reversing antibiotic resistance. Expert Opin Investig Drugs 11(8):1023–1032. 10.1517/13543784.11.8.1023 12150699 10.1517/13543784.11.8.1023

[CR19] Jenks PJ, Edwards DI (2002) Metronidazole resistance in *Helicobacter pylori*. Int J Antimicrob Agents 19(1):1–7. 10.1016/S0924-8579(01)00468-X 11814762 10.1016/s0924-8579(01)00468-x

[CR20] Johnson PJ, Schuck BL, Delgadillo MG (1994) Analysis of a single-domain P-glycoprotein-like gene in the early-diverging protist *Trichomonas vaginalis*. Mol Biochem Parasitol 66:127–137. 10.1016/0166-6851(94)90043-4 7984175 10.1016/0166-6851(94)90043-4

[CR21] Kabra R, Chauhan N, Kumar A, Ingale P, Singh S (2019) Efflux pumps and antimicrobial resistance: paradoxical components in systems genomics. Prog Biophys Mol Biol 141:15–24. 10.1016/0166-6851(94)90043-4 30031023 10.1016/j.pbiomolbio.2018.07.008 PMC7173168

[CR22] Kirkcaldy RD, Augostini P, Asbel LE, Bernstein KT, Kerani RP, Mettenbrink CJ, Pathela P, Schwebke JR, Secor WE, Workowski KA, Davis D (2012) *Trichomonas vaginalis* antimicrobial drug resistance in 6 US cities, STD Surveillance Network, 2009–2010. Emerg Infect Dis 18(6):939. 10.3201/eid1806.111590 22608054 10.3201/eid1806.111590 PMC3358158

[CR23] Kissinger P, Muzny CA, Mena LA, Lillis RA, Schwebke JR, Beauchamps L, Taylor SN, Schmidt N, Myers L, Augostinig P, Secor WE, Bradic M, Carlton JM, Martin DH (2018) Single-dose versus 7-day-dose metronidazole for the treatment of trichomoniasis in women: an open-label, randomised controlled trial. Lancet Infect Dis 18(11):1251–1259. 10.1016/s1473-3099(18)30423-7 30297322 10.1016/S1473-3099(18)30423-7 PMC6279510

[CR24] Kouhsari E, Douraghi M, Krutova M, Yaseri HF, Talebi M, Baseri Z, Moqarabzadeh V, Sholeh M, Amirmozafari N (2019) The emergence of metronidazole and vancomycin reduced susceptibility in *Clostridium difficile* isolates in Iran. J Glob Antimicrob Resist 18:28–33. 10.1016/j.jgar.2019.01.027 30703583 10.1016/j.jgar.2019.01.027

[CR25] Kulda J (1999) Trichomonads, hydrogenosomes and drug resistance. Int J Parasitol 29:199–212. 10.1016/S0020-7519(98)00155-6 10221623 10.1016/s0020-7519(98)00155-6

[CR26] Leitsch D (2019) A review on metronidazole: an old warhorse in antimicrobial chemotherapy. Parasitology 146:1167–1178. 10.1017/S0031182017002025 29166971 10.1017/S0031182017002025

[CR27] Leitsch D, Drinić M, Duchêne M (2012) Down-regulation of flavin reductase and alcohol dehydrogenase-1 (ADH-1) in metronidazole-resistant isolates of *Trichomonas vaginalis*. Mol Biochem Parasitol 183:177–183. 10.1016/j.molbiopara.2012.03.003 22449940 10.1016/j.molbiopara.2012.03.003 PMC3341570

[CR28] Leitsch D, Janssen BD, Kolarich D, Johnson PJ, Duchêne M (2014) *Trichomonas vaginalis* flavin reductase 1 and its role in metronidazole resistance. Mol Microbiol 91:198–208. 10.1111/mmi.12455 24256032 10.1111/mmi.12455 PMC4437529

[CR29] Leitsch D, Kolarich D, Binder M, Stadlmann J, Altmann F, Duchêne M (2009) *Trichomonas vaginalis*: metronidazole and other nitroimidazole drugs are reduced by the flavin enzyme thioredoxin reductase and disrupt the cellular redox system. Implications for nitroimidazole toxicity and resistance. Mol Microbiol 72:518–536. 10.1111/j.1365-2958.2009.06675.x 19415801 10.1111/j.1365-2958.2009.06675.x

[CR30] Leitsch D, Kolarich D, Duchêne M (2010) The flavin inhibitor diphenyleneiodonium renders *Trichomonas vaginalis* resistant to metronidazole, inhibits thioredoxin reductase and flavin reductase, and shuts off hydrogenosomal enzymatic pathways. Mol Biochem Parasitol 171:17–24. 10.1016/j.molbiopara.2010.01.001 20093143 10.1016/j.molbiopara.2010.01.001

[CR31] Linstead DJ, Bradley S (1988) The purification and properties of two soluble reduced nicotinamide: acceptor oxidoreductases from *Trichomonas vaginalis*. Mol Biochem Parasitol 27:125–133. 10.1016/0166-6851(88)90032-1 3257811 10.1016/0166-6851(88)90032-1

[CR32] Lossick JG, Kent HL (1991) Trichomoniasis: trends in diagnosis and management. Am J Obstet Gynecol 165:1217–1222. 10.1016/S0002-9378(12)90730-9 1951578 10.1016/s0002-9378(12)90730-9

[CR33] Mayr AL, Paunkov A, Hummel K, Razzazi-Fazeli E, Leitsch D (2024) Comparative proteomic analysis of metronidazole-sensitive and resistant *Trichomonas vaginalis* suggests a novel mode of metronidazole action and resistance. Int J Parasitol Drugs Drug Resist 100566. 10.1016/j.ijpddr.2024.100566. 10.1016/j.ijpddr.2024.100566 PMC11490683 39368438

[CR34] McClelland RS, Sangare L, Hassan WM, Lavreys L, Mandaliya K, Kiarie J, Ndinya-Achola JO, Baeten JM (2007) Infection with *Trichomonas vaginalis* increases the risk of HIV-1 acquisition. J Infect Dis 195:698–702. 10.1086/511278 17262712 10.1086/511278

[CR35] McNeil CJ, Williamson JC, Muzny CA (2023) Successful treatment of persistent 5-nitroimidazole–resistant trichomoniasis with an extended course of oral secnidazole plus intravaginal boric acid. Sex Transm Dis 50:243–246. 10.1097/OLQ.0000000000001741 36730040 10.1097/OLQ.0000000000001741 PMC10010696

[CR36] Müller M, Lossick JG, Gorrell TE (1988) In vitro susceptibility of *Trichomonas vaginalis* to metronidazole and treatment outcome in vaginal trichomoniasis. Sex Transm Dis 15(1):17–24. 10.1097/00007435-198801000-00004 3258675 10.1097/00007435-198801000-00004

[CR37] Muzny CA, Kissinger PJ (2023) Where do tinidazole and secnidazole fit in with the treatment of trichomoniasis? Sex Transm Dis 50(10):e17–e21. 10.1097/OLQ.0000000000001850 37432997 10.1097/OLQ.0000000000001850

[CR38] Muzny CA, Schwebke JR, Nyirjesy P, Kaufman G, Mena LA, Lazenby GB, Van Gerwen OT, Graves KJ, Arbuckle J, Carter BA, McMahon CP, Eder S, Shaw J, Pandey B, Chavoustie SE (2021) Efficacy and safety of single oral dosage of secnidazole for trichomoniasis in women: results of a phase 3, randomized, double-blind, placebo-controlled, delayed-treatment study. Clin Infect Dis 73(6):e1282–e1289. 10.1093/cid/ciab242 33768237 10.1093/cid/ciab242 PMC8442793

[CR39] Muzny CA, Van Gerwen OT, Legendre D (2022) Secnidazole: a treatment for trichomoniasis in adolescents and adults. Expert Rev Anti Infect Ther 20:1067–1076. 10.1080/14787210.2022.2080656 35642509 10.1080/14787210.2022.2080656 PMC9844242

[CR40] Pal D, Banerjee S, Cui J, Schwartz A, Ghosh SK, Samuelson J (2009) *Giardia*, *Entamoeba*, and *Trichomonas* enzymes activate metronidazole (nitroreductases) and inactivate metronidazole (nitroimidazole reductases). Antimicrob Agents Chemother 53:458–464. 10.1128/aac.00909-08 19015349 10.1128/AAC.00909-08 PMC2630645

[CR41] Paulish-Miller TE, Augostini P, Schuyler JA, Smith WL, Mordechai E, Adelson ME, Gygax SE, Secor WE, Hilbert DW (2014) *Trichomonas vaginalis* metronidazole resistance is associated with single nucleotide polymorphisms in the nitroreductase genes *ntr4Tv* and *ntr6Tv*. Antimicrob Agents Chemother 58:2938–2943. 10.1128/aac.02370-13 24550324 10.1128/AAC.02370-13 PMC3993245

[CR42] Paunkov A, Kupc M, Sóki J, Leitsch D (2022) Characterization of the components of the thioredoxin system in *Bacteroides fragilis* and evaluation of its activity during oxidative stress. Anaerobe 73:102507. 10.1016/j.anaerobe.2021.102507 34979246 10.1016/j.anaerobe.2021.102507

[CR43] Pérez-Victoria JM, Cortés-Selva F, Parodi-Talice A, Bavchvarov BI, Pérez-Victoria FJ, Muñoz-Martínez F, Maitrejean M, Costi MP, Barron D, Di Pietro A, Castanys S, Gamarro F (2006) Combination of suboptimal doses of inhibitors targeting different domains of *LtrMDR1* efficiently overcomes resistance of *Leishmania* spp. to miltefosine by inhibiting drug efflux. Antimicrob Agents Chemother 50:3102–3110. 10.1128/aac.00423-06 16940108 10.1128/AAC.00423-06 PMC1563564

[CR44] Petrin D, Delgaty K, Bhatt R, Garber G (1998) Clinical and microbiological aspects of *Trichomonas vaginalis*. Clin Microbiol Rev 11:300–317. 10.1128/cmr.11.2.300 9564565 10.1128/cmr.11.2.300 PMC106834

[CR45] Robinson SC (1962) Trichomonal vaginitis resistant to metronidazole. Can Med Assoc J 86:665 20327097 PMC1849337

[CR46] Roe FJC (1985) Safety of nitroimidazoles. Scand J Infect Dis Suppl 46:64–71. 10.3109/inf.1985.17.suppl-46.01 3865353

[CR47] Rowley J, Vander Hoorn S, Korenromp E, Low N, Unemo M, Abu-Raddad LJ, Chico RM, Smolak A, Newman L, Gottlieb S, Thwin SS, Broutet N, Taylor MM (2019) Chlamydia, gonorrhoea, trichomoniasis and syphilis: global prevalence and incidence estimates. Bull World Health Organ 97:548-562P. 10.2471/BLT.18.228486 31384073 10.2471/BLT.18.228486 PMC6653813

[CR48] RStudio Team (2024) RStudio: integrated development for R. RStudio, PBC, Boston, MA. Retrieved from http://www.rstudio.com/.

[CR49] Saurina G, DeMeo L, McCormack WM (1998) Cure of metronidazole- and tinidazole-resistant trichomoniasis with use of high-dose oral and intravaginal tinidazole. Clin Infect Dis 26:1238–1239. 10.1086/598357 9597267 10.1086/598357

[CR50] Sawyer PR, Brogden RN, Pinder RM, Speight TM, Avery GS (1996) Tinidazole: a review of its antiprotozoal activity and therapeutic efficacy. Drugs 11:423–440. 10.2165/00003495-197611060-00003 10.2165/00003495-197611060-00003 954609

[CR51] Shemenski J (2019) Cimetidine as a novel adjunctive treatment for early-stage Lyme disease. Med Hypotheses 128:94–100. 10.1016/j.mehy.2016.03.015 27107653 10.1016/j.mehy.2016.03.015

[CR52] Shukla S, Ohnuma S, Ambudkar SV (2011) Improving cancer chemotherapy with modulators of ABC drug transporters. Curr Drug Targets 12:621–630. 10.2174/138945011795378540 21039338 10.2174/138945011795378540 PMC3401946

[CR53] Sobel JD, Nyirjesy P, Brown W (2001) Tinidazole therapy for metronidazole-resistant vaginal trichomonosis. Clin Infect Dis 33:1341–1346. 10.1086/323034 11565074 10.1086/323034

[CR54] Sommer U, Costello CE, Hayes GR, Gilbert RO, Beach DH (2005) Identification of *Trichomonas vaginalis* cysteine proteases that induce apoptosis in human vaginal epithelial cells. J Biol Chem 280:23853–23860. 10.1074/jbc.M501752200 15843376 10.1074/jbc.M501752200

[CR55] Sutcliffe S, Neace C, Magnuson NS, Reeves R, Alderete JF (2012) Trichomonosis, a common curable STI, and prostate carcinogenesis—a proposed molecular mechanism. PLOS Pathog 8:e1002801. 10.1371/journal.ppat.1002801 22912571 10.1371/journal.ppat.1002801 PMC3415452

[CR56] Sutton MY, Sternberg M, Nsuami M et al (1999) Trichomonosis in pregnant human immunodeficiency virus-infected and human immunodeficiency virus-uninfected Congolese women: prevalence, risk factors, and association with low birth weight. Am J Obstet Gynecol 181:656–666. 10.1016/S0002-9378(99)70509-0 10486480 10.1016/s0002-9378(99)70509-0

[CR57] Tang R, Faussat AM, Perrot JY, Marjanovic Z, Cohen S, Storme T, Morjani H, Legrand O, Marie JP (2008) Zosuquidar restores drug sensitivity in P-glycoprotein expressing acute myeloid leukemia (AML). BMC Cancer 8:1–9. 10.1186/1471-2407-8-51 18271955 10.1186/1471-2407-8-51 PMC2258302

[CR58] Twu O, Dessí D, Vu A, Mercer F, Stevens GC, De Miguel N, Rappelli P, Cocco AR, Clubb RT, Fiori PL, Johnson PJ (2014) *Trichomonas vaginalis* homolog of macrophage migration inhibitory factor induces prostate cell growth, invasiveness, and inflammatory responses. Proc Natl Acad Sci 111:8179–8184. 10.1073/pnas.1321884111 24843155 10.1073/pnas.1321884111 PMC4050605

[CR59] Upcroft JA, Dunn L, Wal T, Tabrizi S, Delgadillo-Correa MG, Johnson PJ, Garland S, Siba P, Upcroft P (2009) Metronidazole resistance in *Trichomonas vaginalis* from highland women in Papua New Guinea. Sex Health 6:334–338. 10.1071/SH09011 19917203 10.1071/SH09011

[CR60] Upcroft JA, Dunn LA, Upcroft P (2003) *Trichomonas vaginalis* molecular biology, pharmacology, and drug resistance. In: Fidel PL, Cole RA (eds) Molecular and Clinical Aspects of *Trichomonas Vaginalis*. Springer, pp 177–197

[CR61] Upcroft P, Upcroft JA (2001) Drug targets and mechanisms of resistance in the anaerobic protozoa. Clin Microbiol Rev 14:150–164. 10.1128/cmr.14.1.150-164.2001 11148007 10.1128/CMR.14.1.150-164.2001 PMC88967

[CR62] Voolmann T, Boreham P (1993) Metronidazole-resistant *Trichomonas vaginalis* in Brisbane. Med J Aust 159:490. 10.5694/j.1326-5377.1993.tb137978.x 8412928 10.5694/j.1326-5377.1993.tb137978.x

[CR63] Watt L, Jennison RF (1960) Clinical evaluation of metronidazole. A new systemic trichomonacide. Br Med J 2:902–905. 10.1136/bmj.2.5203.902 13843155 10.1136/bmj.2.5203.902 PMC2098118

[CR64] Wickham H (2016) ggplot2: elegant graphics for data analysis. Springer-Verlag, New York. Retrieved from https://ggplot2.tidyverse.org.

[CR65] Workowski KA (2021) Sexually transmitted infections treatment guidelines, 2021. MMWR Recomm Rep 70. 10.15585/mmwr.rr7004a1 10.15585/mmwr.rr7004a1 PMC8344968 34292926

[CR66] Workowski KA, Bolan GA, Centers for Disease Control and Prevention (2015) Sexually transmitted diseases treatment guidelines, 2015. MMWR Recomm Rep 64(RR-03):1–137. Erratum in: MMWR Recomm Rep 2015 Aug 28;64(33):924. PMC5885289 26042815

[CR67] Yarlett N, Yarlett NC, Lloyd D (1986a) Ferredoxin-dependent reduction of nitroimidazole derivatives in drug-resistant and susceptible strains of *Trichomonas vaginalis*. Biochem Pharmacol 35:1703–1708. 10.1016/0006-2952(86)90327-8 3486660 10.1016/0006-2952(86)90327-8

[CR68] Yarlett N, Yarlett NC, Lloyd D (1986b) Metronidazole-resistant clinical isolates of *Trichomonas vaginalis* have lowered oxygen affinities. Mol Biochem Parasitol 19:111–116. 10.1016/0166-6851(86)90115-5 3487729 10.1016/0166-6851(86)90115-5

