# Supplementary material for: Roles of efflux pumps and nitroreductases in metronidazole-resistant Trichomonas vaginalis
Source: Parasitol Res. 2025 Feb 12;124(2):21. doi: 10.1007/s00436-025-08463-7 (PMC11821713; doi:10.1007/s00436-025-08463-7)
Supplement: Supplementary file 1 — Supplementary file1 (DOCX 127 KB) [file 436_2025_8463_MOESM1_ESM.docx]

| 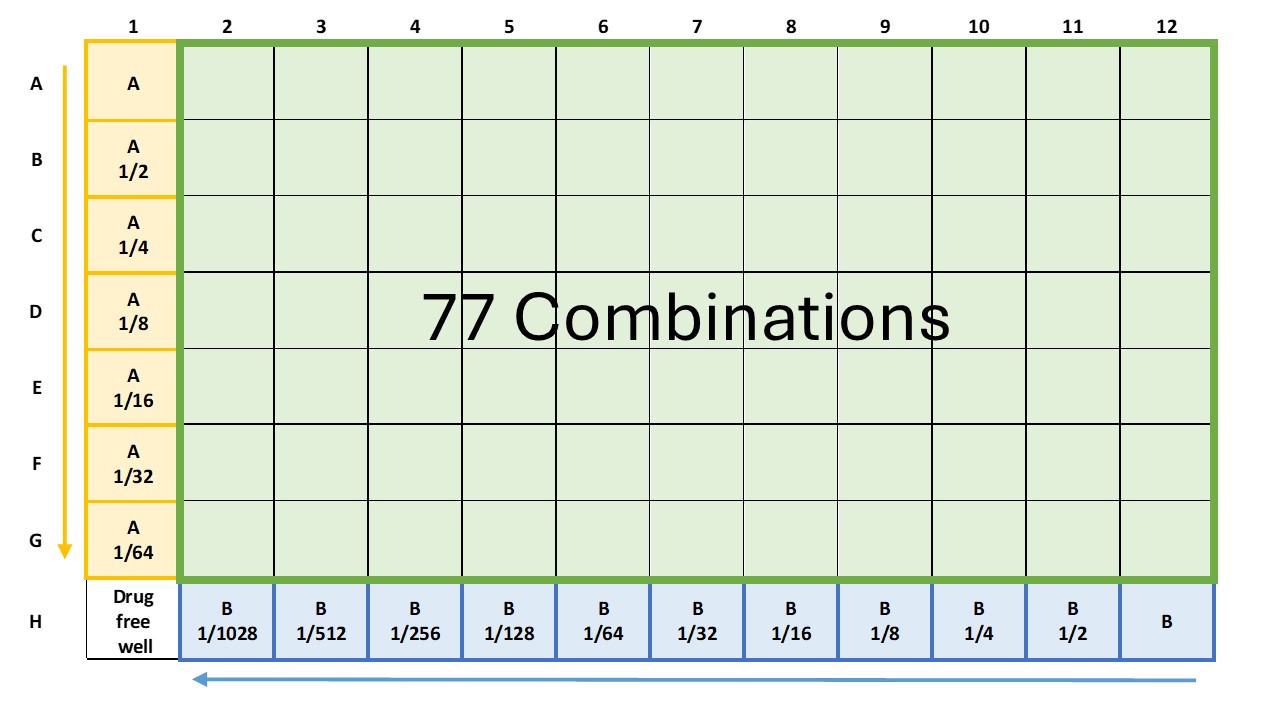 |
| --- |
| **Supplementary figure 1.** The 96-well plate is organized to evaluate the interactions between two drugs using a checkerboard assay. In column 1, from wells A1 to G1, double serial dilutions of drug A (either metronidazole or tinidazole) are arranged, with the highest concentration of drug A in well A1 and each subsequent well down the column representing a twofold dilution. Row H, from wells H12 to H2, contains double serial dilutions of drug B (efflux inhibitors), with the highest concentration in well H12 and each subsequent well to the left representing a twofold dilution. Well H1 serves as the growth control without drugs. The remaining wells of the plate represent all 77 possible combinations formed by the interaction of the serial dilutions of each drug. |
