# Supplementary material for: Roles of efflux pumps and nitroreductases in metronidazole-resistant Trichomonas vaginalis
Source: Parasitol Res. 2025 Feb 12;124(2):21. doi: 10.1007/s00436-025-08463-7 (PMC11821713; doi:10.1007/s00436-025-08463-7)
Supplement: Supplementary file 2 — Supplementary file2 (DOCX 57 KB) [file 436_2025_8463_MOESM2_ESM.docx]

| **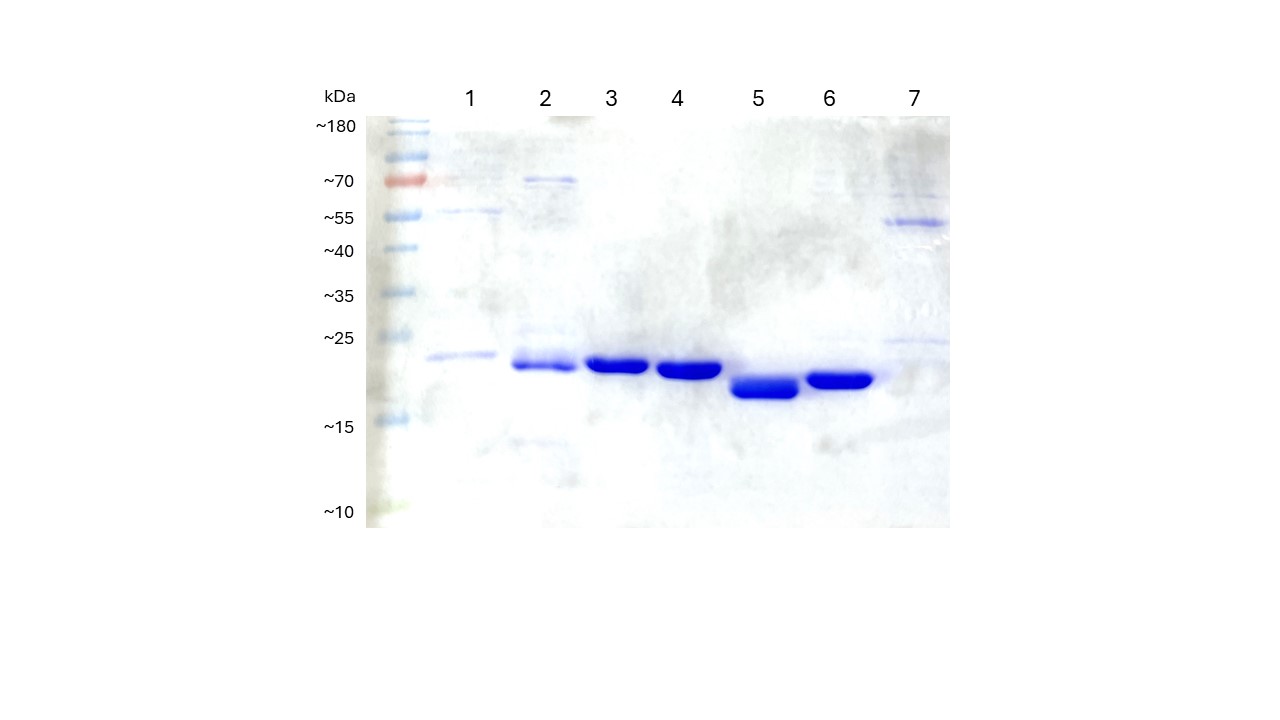** |
| --- |
| **Supplementary figure 2.** 10µg of purified recTvNtr1 (1), recTvNtr4 (2), recTvNtr6 (3), recTvNtr8 (4), recTvNtr9 (5), recTvNtr10 (6) and recTvOxR1 (7) alongside the 5µL of PageRuler (Thermo Fisher Scientific) on 12.5% SDS-PAGE gel stained with Coomassie brilliant blue. |
