# Supplementary material for: Roles of efflux pumps and nitroreductases in metronidazole-resistant Trichomonas vaginalis
Source: Parasitol Res. 2025 Feb 12;124(2):21. doi: 10.1007/s00436-025-08463-7 (PMC11821713; doi:10.1007/s00436-025-08463-7)
Supplement: Supplementary file 3 — Supplementary file3 (DOCX 14 KB) [file 436_2025_8463_MOESM3_ESM.docx]

**Ntr7**

**Data base entry NCBI/Genbank:** EAY04224/ XP_001316447

**Database entry TrichoDB:** TVAG_474290

**Original sequence with NdeI restriction site:**

ATGTTTGACGCAATCAAAGAAAGACGTGCTGTTAGATCTTACATTACAGAAGAGAAAATCCCAAAGGATGTTCTTGAAAATATTATTAAAGCAGCTCAAAATGCTCCAACAGGATGCGATTTCCAATCATATGATTTCATAGTTGATACTAACAGAGAACATCTTACTAAGATTGCACACGCAGTCTATAACTCATTCCCAGGCATCGATAAGTTCCTTAAGAATCCAGATATCTTCTATGGTGCTCCATGCGTTATTTTCATTGTACCTGCACGTCCATTCCGTGAAGACTGCTGCGTATATGATATGGGCATTATCGGTCAGTCCATTTGCTTAGAAGCTAAAGTTCAAGGTTATGCTTCAGTTCAAATTGGATTCGTCCATGGAACTAAACCAGAAGTCCTTAAACCATTCCTTGATTTGCCACGCGATTTGAGCCCATTAGCTGTTGCAATTGGAAAGCCTTCACCTGATTTCACACCAGCACCAAAGGAAATCACATCCAAGATTCACTGA

**New sequence with mutation introduced in NdeI restriction site:**

ATGTTTGACGCAATCAAAGAAAGACGTGCTGTTAGATCTTACATTACAGAAGAGAAAATCCCAAAGGATGTTCTTGAAAATATTATTAAAGCAGCTCAAAATGCTCCAACAGGATGCGATTTCCAATCATA**C**GATTTCATAGTTGATACTAACAGAGAACATCTTACTAAGATTGCACACGCAGTCTATAACTCATTCCCAGGCATCGATAAGTTCCTTAAGAATCCAGATATCTTCTATGGTGCTCCATGCGTTATTTTCATTGTACCTGCACGTCCATTCCGTGAAGACTGCTGCGTATATGATATGGGCATTATCGGTCAGTCCATTTGCTTAGAAGCTAAAGTTCAAGGTTATGCTTCAGTTCAAATTGGATTCGTCCATGGAACTAAACCAGAAGTCCTTAAACCATTCCTTGATTTGCCACGCGATTTGAGCCCATTAGCTGTTGCAATTGGAAAGCCTTCACCTGATTTCACACCAGCACCAAAGGAAATCACATCCAAGATTCACTGA

**Final sequence with Ndel and XhoI restriction sites and 6x His tag:**

CATATGTTTGACGCAATCAAAGAAAGACGTGCTGTTAGATCTTACATTACAGAAGAGAAAATCCCAAAGGATGTTCTTGAAAATATTATTAAAGCAGCTCAAAATGCTCCAACAGGATGCGATTTCCAATCATA**C**GATTTCATAGTTGATACTAACAGAGAACATCTTACTAAGATTGCACACGCAGTCTATAACTCATTCCCAGGCATCGATAAGTTCCTTAAGAATCCAGATATCTTCTATGGTGCTCCATGCGTTATTTTCATTGTACCTGCACGTCCATTCCGTGAAGACTGCTGCGTATATGATATGGGCATTATCGGTCAGTCCATTTGCTTAGAAGCTAAAGTTCAAGGTTATGCTTCAGTTCAAATTGGATTCGTCCATGGAACTAAACCAGAAGTCCTTAAACCATTCCTTGATTTGCCACGCGATTTGAGCCCATTAGCTGTTGCAATTGGAAAGCCTTCACCTGATTTCACACCAGCACCAAAGGAAATCACATCCAAGATTCACCATCACCATCACCATCACTGACTCGAG

**Ntr11**

**Data base entry NCBI/Genbank:** EAY14310/ XP_001326533

**Database entry TrichoDB:** TVAG_026310

**Original sequence with NdeI restriction site:**

ATGACAAGCGTGTTTGAATGTATAGAAAGAAGAAGAACTATTAGGCATTATGATCAAAATTGGGTATGTCCAAAAGAGCACCTTGAAGCTATTGTAAATGCAGCTCTAAAATCCCCAACTGCATGTAATCGTCAAAGTATTGACCTTCTCGTTATTACAAACAAAGAGGTATTGGATAAAATAGGTGAAGTTGGTCTTAATACATTAAAGAAAGGTACAAAAGAGCATATGGAAGAAAGAAAACATGAAGGATACAAAAATGTTTTTACTTGTGATGCTCCTGTGCTCTTTCTTTTGGTTAAAAACGATAGGGTTAATCCATTATATACAGATGTCGATGCTGGAATCATGTGCGAATCAATTATGCTCACTGCAGCAAGTTACGGATATGGAACAATGTGCATTGGTGTTCTAAGAGCAACTGACTTATATGAAGCTGTAGGAATTCACAAAGAAGACCTTGCAATGGCTGTTTGCATGGGTAAAATCGAAGATGGATACGTTCCACCGGAAAAACCAATCAAATGCAAAGCCACATACATAGAATAA

**New sequence with mutation introduced in NdeI restriction site:**

ATGACAAGCGTGTTTGAATGTATAGAAAGAAGAAGAACTATTAGGCATTATGATCAAAATTGGGTATGTCCAAAAGAGCACCTTGAAGCTATTGTAAATGCAGCTCTAAAATCCCCAACTGCATGTAATCGTCAAAGTATTGACCTTCTCGTTATTACAAACAAAGAGGTATTGGATAAAATAGGTGAAGTTGGTCTTAATACATTAAAGAAAGGTACAAAAGAGCA**C**ATGGAAGAAAGAAAACATGAAGGATACAAAAATGTTTTTACTTGTGATGCTCCTGTGCTCTTTCTTTTGGTTAAAAACGATAGGGTTAATCCATTATATACAGATGTCGATGCTGGAATCATGTGCGAATCAATTATGCTCACTGCAGCAAGTTACGGATATGGAACAATGTGCATTGGTGTTCTAAGAGCAACTGACTTATATGAAGCTGTAGGAATTCACAAAGAAGACCTTGCAATGGCTGTTTGCATGGGTAAAATCGAAGATGGATACGTTCCACCGGAAAAACCAATCAAATGCAAAGCCACATACATAGAATAA

**Final sequence with Ndel and XhoI restriction sites and 6x His tag:**

CATATGACAAGCGTGTTTGAATGTATAGAAAGAAGAAGAACTATTAGGCATTATGATCAAAATTGGGTATGTCCAAAAGAGCACCTTGAAGCTATTGTAAATGCAGCTCTAAAATCCCCAACTGCATGTAATCGTCAAAGTATTGACCTTCTCGTTATTACAAACAAAGAGGTATTGGATAAAATAGGTGAAGTTGGTCTTAATACATTAAAGAAAGGTACAAAAGAGCA**C**ATGGAAGAAAGAAAACATGAAGGATACAAAAATGTTTTTACTTGTGATGCTCCTGTGCTCTTTCTTTTGGTTAAAAACGATAGGGTTAATCCATTATATACAGATGTCGATGCTGGAATCATGTGCGAATCAATTATGCTCACTGCAGCAAGTTACGGATATGGAACAATGTGCATTGGTGTTCTAAGAGCAACTGACTTATATGAAGCTGTAGGAATTCACAAAGAAGACCTTGCAATGGCTGTTTGCATGGGTAAAATCGAAGATGGATACGTTCCACCGGAAAAACCAATCAAATGCAAAGCCACATACATAGAACATCACCATCACCATCACTAACTCGAG

**Ntr-like 3**

**Data base entry NCBI/Genbank:** EAY07485/XP_001319708

**Database entry TrichoDB:**  TVAG_499730

**Original sequence with NdeI restriction site:**

ATGTCGAGTGAACAAGCCTCACATATGAGCTTCTATGAAGCAGTTGAAAAGAGAAGAATGGTCTGTGACTTCCAAGATAAAGAAGTTCCAGAAGCTGTCTTGAAAAGAATTATTGATGCAGGTTTAAAGGCTCCAACATATGATCACCTCAGAAACTGGGAATTTATCATAGTGAAAGATCCTAAGGACAAGAAGCATGCTCTTCAATTCATTGAGCAATCAACACCTGCTCAGCTCAAGATTTTGGAAGAAACATTATCTAAAGGCTCAGCACAGGAGAAGATGTATTCAATTGCTATGCCAAGACAATATTCCATGCTTTATAATGCATCATATATTGTTTTTCCATTCTTCAAATCCACACCAGATCTCATGCATCCAACATGCGTCAGTTCATTAAACCCAATTTCCTCTATTTGGTGTGTAATTGAGAACATTTTCCTTGCTGCAACTGCTGAAGGTTTGGCCTGCTCTATGAGAATCCCTGTTGGAGAAGAAGGACCAAATGTCGCAAAGGCTATTGGCGCACCAGATGACTACCTCTTGCCATGCTATTTGGGAATTGGATATCCTTTGGAAGACAGACCAAAGATTGAGCAAATCCAATTCACAGCCGAACAAAAGGTGCACTATGGCAAATGGTAA

**New sequence with mutation introduced in NdeI restriction site:**

ATGTCGAGTGAACAAGCCTCACATATGAGCTTCTATGAAGCAGTTGAAAAGAGAAGAATGGTCTGTGACTTCCAAGATAAAGAAGTTCCAGAAGCTGTCTTGAAAAGAATTATTGATGCAGGTTTAAAGGCTCCAACATA**C**GATCACCTCAGAAACTGGGAATTTATCATAGTGAAAGATCCTAAGGACAAGAAGCATGCTCTTCAATTCATTGAGCAATCAACACCTGCTCAGCTCAAGATTTTGGAAGAAACATTATCTAAAGGCTCAGCACAGGAGAAGATGTATTCAATTGCTATGCCAAGACAATATTCCATGCTTTATAATGCATCATATATTGTTTTTCCATTCTTCAAATCCACACCAGATCTCATGCATCCAACATGCGTCAGTTCATTAAACCCAATTTCCTCTATTTGGTGTGTAATTGAGAACATTTTCCTTGCTGCAACTGCTGAAGGTTTGGCCTGCTCTATGAGAATCCCTGTTGGAGAAGAAGGACCAAATGTCGCAAAGGCTATTGGCGCACCAGATGACTACCTCTTGCCATGCTATTTGGGAATTGGATATCCTTTGGAAGACAGACCAAAGATTGAGCAAATCCAATTCACAGCCGAACAAAAGGTGCACTATGGCAAATGGTAA

**Final sequence with Ndel and XhoI restriction sites and 6x His tag:**

CATATGTCGAGTGAACAAGCCTCACATATGAGCTTCTATGAAGCAGTTGAAAAGAGAAGAATGGTCTGTGACTTCCAAGATAAAGAAGTTCCAGAAGCTGTCTTGAAAAGAATTATTGATGCAGGTTTAAAGGCTCCAACATA**C**GATCACCTCAGAAACTGGGAATTTATCATAGTGAAAGATCCTAAGGACAAGAAGCATGCTCTTCAATTCATTGAGCAATCAACACCTGCTCAGCTCAAGATTTTGGAAGAAACATTATCTAAAGGCTCAGCACAGGAGAAGATGTATTCAATTGCTATGCCAAGACAATATTCCATGCTTTATAATGCATCATATATTGTTTTTCCATTCTTCAAATCCACACCAGATCTCATGCATCCAACATGCGTCAGTTCATTAAACCCAATTTCCTCTATTTGGTGTGTAATTGAGAACATTTTCCTTGCTGCAACTGCTGAAGGTTTGGCCTGCTCTATGAGAATCCCTGTTGGAGAAGAAGGACCAAATGTCGCAAAGGCTATTGGCGCACCAGATGACTACCTCTTGCCATGCTATTTGGGAATTGGATATCCTTTGGAAGACAGACCAAAGATTGAGCAAATCCAATTCACAGCCGAACAAAAGGTGCACTATGGCAAATGGCATCACCATCACCATCACTAACTCGAG
