# Supplementary material for: Roles of efflux pumps and nitroreductases in metronidazole-resistant Trichomonas vaginalis
Source: Parasitol Res. 2025 Feb 12;124(2):21. doi: 10.1007/s00436-025-08463-7 (PMC11821713; doi:10.1007/s00436-025-08463-7)
Supplement: Supplementary file 4 — Supplementary file4 (DOCX 20 KB) [file 436_2025_8463_MOESM4_ESM.docx]

| **Name** | **Data base entry NCBI/Genbank** | **Database entry TrichoDB** | **Size: bp, kDa and amino acid (AA)** | **Primer sequences** |
| --- | --- | --- | --- | --- |
| **Ntr1** | EAX85619/ XP_001298549 | TVAG_036500 | 540bp (19.98kDa = 180AA) | Forward: GACATATGGCAATTTCTGCTATCGAAGCTT  Reverse: GACTCGAGTTAGTGATGGTGATGGTGATGTTCTATATAAAAAATCTTTCTTAAGTTAGGCTT |
| **Ntr2** | EAX89456/ XP_001302386 | TVAG_455650 | 594bp (21.97kDa = 198AA) | Forward: GACATATGCATTCACAAATAAAAATCAGTTCA  Reverse: GACTCGAGTTAGTGATGGTGATGGTGATGGTAATAGTGGACCTTAGCTTTGA |
| **Ntr3** | EAX89567/ XP_001302497 | TVAG_356820 | 549bp (20.31kDa = 183AA) | Forward: GACATATGAGTGTTCTCAAGTGCAT  Reverse: GACTCGAGTTAGTGATGGTGATGGTGATGGTCAATATGAGTAATCTTTCCCTT |
| **Ntr4** | EAX94976/ XP_001307906 | TVAG_205740 | 549bp (20.31kDa = 183AA) | Forward: GACATATGAGTGTCCTTAAGTGCATCCA  Reverse: GACTCGAGTTAGTGATGGTGATGGTGATGGTCGGCATAAACTACCTTAGAC |
| **Ntr5** | EAX96000/XP_001308930 | TVAG_052580 | 543bp (20.091kDa = 181AA) | Forward: GACATATGTCCGTTTTTGATGCTATTGA  Reverse: GACTCGAGTTAGTGATGGTGATGGTGATGTTCTAAGTAAGTTATCTTTGTATTTC |
| **Ntr6** | EAX95789/ XP_001308719 | TVAG_354010 | 543bp (20.091kDa = 181AA) | Forward: GACATATGTCTATCTCACAACTCAAGTCC  Reverse: GACTCGAGTTAGTGATGGTGATGGTGATG TTCAATGTATGTAACCTTTCTAAG |
| **Ntr8** | EAY05392/ XP_001317615 | TVAG_131260 | 546bp (20.202kDa = 182AA) | Forward: GACATATGGCTCTTGCGGCTCTCA  Reverse: GACTCGAGTTAGTGATGGTGATGGTGATGCTCTGGAACGAACTGAACCTT |
| **Ntr9** | EAY15872/ XP_001328095 | TVAG_165000 | 546bp (20.202kDa = 163AA) | Forward: GACATATGAACGTGCTTGATGCGATTT  Reverse: GACTCGAGTTAGTGATGGTGATGGTGATGTTCTATAAATGATGCCTTGCAAAG |
| **Ntr10** | EAY16021/XP_001328244 | TVAG_277870 | 543bp (20.091kDa = 182AA) | Forward: GACATATGGCTCTTGAAGTCCTTAAGGC  Reverse: GACTCGAGTTAGTGATGGTGATGGTGATGTTCAAGGAATGTAACTTTACGGAGAACC |
| **Ntr-like 1** | EAY17166/XP_001329389 | TVAG_303860 | 561bp (20.757kDa = 187AA) | Forward: GACATATGGATGTTCTTTTAAAGAGACGCTCTG  Reverse: GACTCGAGTTAGTGATGGTGATGGTGATGTTCAATGTATTTAACGTTAGACTTAATCTCCT |
| **Ntr-like 2** | EAX93690/XP_001306620 | TVAG_473580 | 831bp (30.747kDa = 277AA) | Forward: GACATATGTTAGAAGTGAAGGAATTTTCACTCATT  Reverse: GACTCGAGTTAGTGATGGTGATGGTGATGAAATACGCCTTCTTCAACATAAATCGTG |
| **Oxidoreductase 1** | EAY14856/XP_001327079 | TVAG_411220 | 1008bp (37.296kDa = 336AA) | Forward: GACATATG TTAGACAGATTGTTTACTCCAGTGA  Reverse: GACTCGAGTTAGTGATGGTGATGGTGATGAAATGCATAGCATTTGATTGGTTGT |
| **Oxidoreductase 2** | EAY11058/ XP_001323281 | TVAG_044820 | 627bp (23.199kDa = 209AA) | Forward: GACATATGCTTGTCCACTGGCCAG  Reverse: GACTCGAGTTAGTGATGGTGATGGTGATG GTGACCAAGGTTAAGAGCATC |
| **Oxidoreductase 3** | EAY09752 /XP_001321975 | TVAG_414030 | 969bp (35.853kDa = 323AA) | Forward: GACATATGAATTTAGGTGTTCTCGGTACTGGC  Reverse: GACTCGAGTTAGTGATGGTGATGGTGATGAACTTTGATGCCAAGTTGTCTTC |

**Supplementary table 1.** Primer sequences of nitroreductases, nitroreductase-like proteins and oxidoreductases.
